# Evaluation of Macrophage Activation Syndrome in Patients with Systemic Juvenile Idiopathic Arthritis: A Single Center Experience

**DOI:** 10.1155/2022/1784529

**Published:** 2022-07-27

**Authors:** Pia Elkjær Høeg, Mia Glerup, Birgitte Mahler, Christian Høst, Troels Herlin

**Affiliations:** ^1^Pediatric Rheumatology Clinic, Department of Pediatrics and Adolescent Medicine, Aarhus University Hospital, Denmark; ^2^Diagnostic Center, Regional Hospital Silkeborg, Silkeborg, Denmark

## Abstract

**Objectives:**

Macrophage activation syndrome (MAS) is a severe complication of systemic juvenile arthritis (sJIA), and early diagnosis is critical for survival. The objective of this study was to evaluate the 2016 MAS classification criteria in a Danish sJIA cohort and to compare different sets of criteria for the early identification of MAS including the HLH-2004 diagnostic guidelines, MS score, and the ferritin/ESR ratio.

**Methods:**

Data was extracted from medical charts of 32 patients with sJIA from a single Danish paediatric rheumatology center diagnosed between January 2014 and June 2021. Patients who met the 2016 MAS classification criteria were classified as having MAS. From a receiver operating characteristic (ROC) plot, the area under the curve (AUC) was calculated for the prediction of patients with MAS according to the 2016 MAS classification criteria using either MS score or the ferritin/ESR ratio.

**Results:**

Of the cohort, eight (25%) patients were classified as having MAS according to the 2016 MAS classification criteria compared to only three (9.4%) patients fulfilling the HLH-2004 diagnostic guidelines, all of which had recurrent MAS. The ferritin/ESR ratio showed the highest sensitivity (100%) but the lowest specificity (72.2%). In comparison, the MS score had a higher specificity (90.9%) for the identification of MAS according to the 2016 classification criteria. In our cohort, the most optimal cut-off point for the ferritin/ESR ratio was ≥19.4 (sensitivity: 100%, specificity: 72.2%) and ≥ -1.5 for the MS score (sensitivity: 71.4%, specificity: 91.7%), respectively.

**Conclusion:**

The 2016 MAS classification criteria were a valuable tool in the discrimination of sJIA with and without MAS. The HLH-2004 diagnostic guidelines showed the lowest sensitivity, ferritin/ESR ratio, and the lowest specificity compared to the MS score where an acceptable high sensitivity and specificity was found.

## 1. Introduction

Macrophage activation syndrome (MAS) is a potentially life-threatening condition which is most common in patients with systemic juvenile idiopathic arthritis (sJIA) [1, 2]. MAS occurs in other inflammatory diseases including juvenile systemic lupus erythematosus and Kawasaki disease [3]. Systemic JIA is a subcategory of JIA characterized by daily spiking fever and arthritis along with lymphadenopathy, serositis, hepatomegaly, splenomegaly, and maculopapular rash according to the International League of Associations for Rheumatology (ILAR) criteria [4]. MAS has been reported in up to 10% of patients with sJIA, whereas subclinical MAS is assumed to occur in about 30-40% with active sJIA [5, 6]. The pathophysiology of MAS is characterized by an uncontrolled immune response involving impaired NK cell cytolytic function, dysregulation of T-lymphocytes, and macrophages resulting in an undue production of cytokines, a cytokine storm [7]. MAS occurs seemingly spontaneous and can be related to infection or drug therapy [3, 8], but in a number of cases with recurrent episodes of MAS, a genetic component has been demonstrated [9, 10].

MAS is clinically characterized by persistent fever, lymphadenopathy, hepatomegaly, splenomegaly, hemorrhage, and central nervous system dysfunction [11, 12]. Laboratory features involve hyperferritinemia, pancytopenia, elevated liver enzymes, hypofibrinogenemia, hypertriglyceridemia, fibrinolytic coagulopathy, and decreased ESR [3].

Presenting symptoms of MAS often mimic a flare of the underlying systemic inflammatory disease or a systemic infection, and the lack of a single pathognomonic feature is a diagnostic obstacle [6, 13, 14]. Because MAS is a potentially fatal condition, it is important to recognize its clinical and laboratory features early [1].

There have been different diagnostic guidelines of MAS in the last decades, including the hemophagocytic lymphohistiocytosis- (HLH-) 2004 diagnostic guidelines primarily developed to diagnose familial HLH [15], the 2016 classification criteria for MAS complicating systemic JIA [6], the more recent MS score [16], and the ferritin to erythrocyte sedimentation rate ratio (ferritin/ESR ratio) [17]. The 2016 MAS classification criteria were based on expert consensus and analysis of data from 683 patients through a multistep process. These classification criteria include the presence of fever and thresholds of ferritin, platelet count, aspartate aminotransferase (AST), triglycerides, and fibrinogen (Table 1) [6]. The 2016 MAS classification criteria were primarily made for further research and clinical trials. Since then, a study has shown that the criteria may not be sensitive enough to identify MAS during biological treatment and probably need refinement for application in these cases [6, 18].

In 2019, Minoia et al. developed a new scoring system, the MS score, to discriminate sJIA-associated MAS from the flare of sJIA [16]. The MS score evaluation was based on the same cohort as was the 2016 MAS classification criteria, but in contrast involves the clinical features: CNS involvement, hemorrhagic manifestations, and arthritis in addition to fever. Additionally, the MS score does not involve certain laboratory thresholds, but instead laboratory values are multiplied by a coefficient [16] .

Eloseily et al. developed the ferritin/ESR ratio in 2019 as a practical tool for diagnosing MAS in sJIA patients [17]. It is based on the assumption that ferritin increases in case of MAS and that ESR will lower due to fibrinogen degradation. Therefore, the ferritin/ESR ratio is expected to increase in the setting of MAS complicating sJIA.

The objective of this study was to evaluate the 2016 MAS classification criteria for the recognition of early development of MAS in a single center Danish sJIA cohort and to compare the performance of other available criteria for MAS complicating sJIA including the HLH-2004 diagnostic guidelines, the MS score, and the ferritin/ESR ratio.

## 2. Methods

### 2.1. Study Design

This retrospective study is based on a systematic examination of medical charts and laboratory values of patients identified with the ICD-10 code DM082. The patients were diagnosed between January 2014 and June 2021 according to the ILAR classification criteria.

In total, thirty-two patients diagnosed with sJIA were included. Collected data included demographic characteristics, laboratory values, and clinical data relevant for the diagnosis of MAS [19].

Based on the 2016 classification criteria for MAS complicating sJIA, the included patients were selected in two groups: “sJIA with MAS” and “sJIA without MAS.” Since AST is not routinely measured in Danish hospitals, the criterion was substituted by the corresponding liver parameter, alanine aminotransferase (ALT), with normal reference values 10-48 U/L.

For MAS patients, the collected information also included duration of sJIA at MAS onset (calculated from the onset of sJIA symptoms), treatment before MAS, number of MAS episodes, and laboratory tests at two timepoints: (1) last visit before the onset of MAS and (2) onset of MAS (defined as the time when the MAS criteria were met the first time). Laboratory changes over time were collected to identify which parameters would be the most important to monitor in the early detection of MAS.

### 2.2. The Selection of the Time of Blood Samples and Clinical Features

2016 MAS classification criteria: for patients with MAS, the first blood sample after the onset of sJIA that met the MAS classification criteria was selected. If a patient had several episodes of MAS, only the first episode was included. For sJIA patients without MAS, the date of the blood sample was based on the highest level of ferritin, coincident with most other available laboratory values after the onset of sJIA. The MS scores were calculated for all patients with available laboratory and clinical data included in the scoring system. Patients with a MS score ≥ −2.1 were defined as sJIA with MAS as in Minoia et al. [16]. The ferritin/ESR ratio: the first ferritin and the coincident ESR during the hospital admission were selected. The patients were categorized as MAS patients if the ferritin/ESR ratio cut-off point was ≥21.5 according to Eloseily et al. [17]. The clinical features were collected at a time interval up to two weeks before and one week after the chosen blood sample date.

### 2.3. Statistics

Quantitative data, not being normally distributed, are shown as the median and interquartile range (IQR), and comparisons were made by Mann–Whitney *U* test. Dichotomous variables were compared by using Pearson's chi-square test. For comparison of paired laboratory patient data, we used Wilcoxon signed rank test for continuous variables. The analyses of sensitivity and specificity of the 2016 MAS classification criteria were determined by Pearson's chi-square test.

We calculated the area under the curve (AUC) from a receiver operating characteristic (ROC) analysis [20], to evaluate the final models' predictive performance. ROC analyses were made to calculate the best cut-off values to discriminate sJIA with and without MAS by using the Youden test [21]. AUC was determined to compare the ability of both score sets to diagnose sJIA-associated MAS in our cohort. A *p* value < 0.05 was considered statistically significant. The statistic program used for the analyses was SPSS vers. 27.

## 3. Results

Of the thirty-two patients with newly diagnosed sJIA, eight (25%) patients were classified as having MAS according to the 2016 MAS classification criteria. Only three (9%) patients fulfilled the HLH-2004 diagnostic guideline criteria (Table 2), which, however, also experienced recurrent MAS. None of the patients diagnosed before 2016 had clinical MAS. One of the patients (ID15), diagnosed in 2015 before the 2016 MAS classification criteria was available and had subclinical MAS, with a transient increase in ferritin, liver transaminases, and low platelet count. Using the MS score, 19 patients could be evaluated, and 7 (37%) of them had a cut point of ≥-2.1 of which 6 also fulfilled the 2016 MAS criteria resulting in a 75% (95% CI 35-97%) sensitivity and 91% (95% CI =59-100%) specificity of identifying MAS according to the 2016 MAS criteria (Table 2). Initial ferritin/ESR ratio could be evaluated in 26 patients, and an elevated ratio (≥21.5) was found in 13 (50%) patients. All patients fulfilling the 2016 MAS classification criteria had a ferritin/ESR ratio above 21.5, resulting in a 100% (95% CI = 63-100%) sensitivity and 72% (95% CI = 47-90%) specificity of identifying MAS according to the 2016 MAS criteria (Table 2).

The demographic characteristics and laboratory data of patients with or without MAS as of the 2016 criteria are shown in Table 3. We found no difference in age at onset or gender between the two groups. Ferritin, triglycerides, ALT, and lactate dehydrogenase (LDH) were significantly higher in the MAS group compared with the group without MAS. Platelets, white blood cell count, neutrophils, ESR, and fibrinogen were significantly lower in MAS patients. However, CRP and hemoglobin showed no significant difference between the two groups (Table 3). The MS score and the ferritin/ESR ratio, respectively, were significantly higher in the group defined by the 2016 MAS classification criteria (Table 3).

We found no significant differences between the groups in any of the clinical signs, including fever, CNS involvement, hepatomegaly, splenomegaly, lymphadenopathy, and hemorrhage. Except for the presence of fever, lymphadenopathy was the most frequent clinical sign in both groups. Lymphadenopathy was observed in 67% of patients with MAS and in 43% of patients without MAS.

Table 4 shows the laboratory values according to the 2016 MAS classification criteria. Five patients fulfilled two criteria, two patients fulfilled three criteria, and one patient fulfilled all four criteria other than high ferritin. The median duration from the onset of sJIA until 2016 MAS classification criteria was fulfilled which was 32 days.

Changes in laboratory values from the last visit before onset of MAS until 2016 MAS classification criteria were met as shown in Table 5. Data of laboratory values for both time points were available for seven patients with MAS. Median interval between the last visit before the onset of MAS and at MAS onset was 22 days. We saw a significant 4-fold increase in ferritin from the last visit before MAS until the onset of MAS and a significant 45% decrease of platelets as well as a significant decrease in WBC and neutrophil count. It was not possible to evaluate the changes in fibrinogen, LDH, and triglycerides due to the lack of laboratory data before the onset of MAS.

Table 6 shows the sensitivity and specificity of the MAS classification criteria. Since ferritin > 684 *μ*g/L is an obligate MAS criterion, sensitivity was 100% but showed low specificity since ten out of eighteen patients without MAS had elevated ferritin, median 1766 *μ*g/L (range 811-3934, *n* = 10). Platelets ≤ 181 × 10^9^/L demonstrated a high specificity rate at 96% but low sensitivity rate at 50%. Among the patients without MAS, only one patient had platelets below the threshold. The threshold for fibrinogen, triglyceride, and ALT showed acceptable sensitivity (62.5-81.8%) and specificity (75-87.5%).

**Figure 1:**
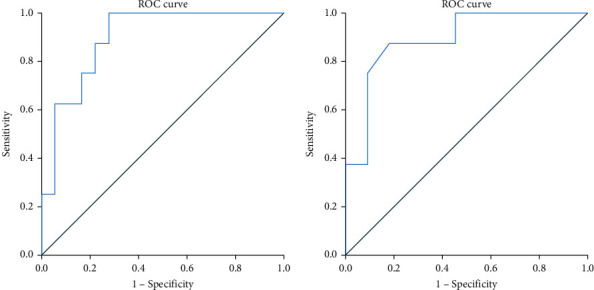
Receiver operating characteristics (ROC) plots illustrating the area under the curve (AUC) model for the prediction of MAS defined by the 2016 MAS classification criteria. (a) Ferritin/ESR ratio *N* = 26, AUC = 0.896 (95% CI: 0.78-1.00), *p* = 0.002. (b) MS score *N* = 19, AUC = 0.892 (95% CI: 0.74-1.00), *p* = 0.004.

From the ROC curves, we calculated the AUCs for the prediction of patients with MAS according to the 2016 classification criteria using either MS score or the ferritin/ESR ratio (Figure 1). The ferritin/ESR ratio varied from 1.17 to 482, AUC being 0.896 (95% CI from 0.78 to 1.00), *p* = 0.002. The MS score varied from -6.7 to 4.3. The AUC of the model was 0.892 with a 95% CI from 0.74 to 1.00, *p* = 0.004. A ferritin/ESR ratio cut-off point of ≥19.4 performed best with a sensitivity rate at 100% and a specificity rate at 72.2%. The optimal cut-off point for the MS score in our cohort was ≥ -1.5 that revealed a sensitivity rate at 71.4% and a specificity rate at 91.7%, respectively.

## 4. Discussion

As of today, there are no diagnostic gold standards for recognizing MAS related to sJIA. The established HLH-2004 guidelines were originally implemented for familial HLH [15] but have not performed well identifying MAS in sJIA, presenting with relative thrombocytosis and leukocytosis [3]. Due to the use of tests not performed in routine labs, the HLH-2004 guidelines may not be practical for the early detection of MAS, which is often crucial for optimal treatment results. The 2016 MAS classification criteria, which were designed for the identification of MAS in patients with sJIA, were based on a large multinational cohort, as were two new scoring tools, the MS score and the ferritin/ESR ratio. This single center study is the first external validation of four different attempts for the early identification of MAS in patients with sJIA.

We found no significant differences in clinical signs between the sJIA groups with or without MAS, indicating that laboratory parameters perform better than the clinical in differentiating between the two groups. Among the recorded clinical signs, lymphadenopathy was the most frequent, but it was not significantly different between the two groups. This finding is in accordance with a study by Kostik et al. who also found comparable presence of lymphadenopathy between patients with and without MAS in a total of 58 patients [2]. Kelly and Ramanan evaluated 95 sJIA patients with MAS and 296 sJIA patients without MAS. They found significant differences in clinical manifestations including a higher percentage of MAS patients presenting with hepatomegaly, splenomegaly, lymphadenopathy, central nervous system dysfunction, and hemorrhage compared to patients not classified with MAS [7]. Clinical signs, however, are often delayed, unspecific, and imitated by other conditions and evidently indicate a progressed course of MAS. For this reason, clinical features (except fever) may not play an essential role in the early diagnosis of MAS [1, 2], which is in accordance with the present study, where the clinical features were collected at a time interval up to two weeks before and one week after the blood sample date. The short duration of median one month between the onset of sJIA and MAS emphasizes the great importance of close disease monitoring in newly diagnosed sJIA patients.

As expected, we found significantly higher levels of ferritin, triglycerides, ALT, and LDH in patients with MAS compared to the group without MAS. In addition, the levels of platelets and fibrinogen were significantly lower in MAS patients. These findings are comparable with Kostik et al. who found similar differences in patients with active sJIA with and without MAS [2]. Zeng et al. collected clinical and laboratory data from thirteen sJIA patients with MAS and found elevated levels of triglycerides (4.2 mmol/L), ALT (153.6 U/L), and LDH (521 U/L) [22].

Ferritin may be a key parameter in the diagnostics of MAS, demonstrating a 4-fold increase from the last visit before MAS until the onset of MAS. It is relevant to notice that patients with active sJIA often have increased ferritin, elevated fibrinogen, and high platelet count as part of their underlying inflammatory response [3, 5]. Even though ferritin > 684 *μ*g/L is an obligate criteria for a diagnosis of MAS according to the 2016 MAS classification criteria, 56% of the patients in the present study without MAS had levels of ferritin above that threshold. Thus, a threshold of 684 *μ*g/L does not exclusively discriminate MAS from a sJIA flare, and likewise, the ferritin/ESR ratio showed low specificity. Being an acute-phase reactant, ferritin is regulated by proinflammatory cytokines (IL-6, IL-18) [23], which levels increase during infection, cancer, and inflammatory conditions such as rheumatologic diseases [24, 25]. Platelets ≤ 181 × 10^9^/L and ALT > 48 U/L showed significant specificity rates of 96% and 87.5%, respectively, which might conceivably be the best laboratory tests in hyperferritinemic patients differentiating between flares of sJIA and MAS. Since AST, ALT, and triglycerides in general are normal in patients with sJIA, a simple increase above the upper limits of these parameters in combination with other criteria and clinical signs may be adequate to indicate the development of MAS [6]. In the preliminary diagnostic guidelines, Ravelli et al. found that platelets ≤ 262 × 10^9^/L among twelve different laboratory features showed the best threshold with a sensitivity of 100% and a specificity of 92% [1]. An absolute number of platelets will probably fail in the diagnosis of MAS in active sJIA patients, since thrombocytosis is a frequent finding in active sJIA as a result of cytokine production [1, 6]. Therefore, a relative decrease of platelets would be preferable to an absolute decrease.

Comparison of the different diagnostic guidelines for MAS showed that the ferritin/ESR ratio had the highest sensitivity but the lowest specificity. In our study, the best cut-off value of the ferritin/ESR ratio was ≥19.4 according to the Youden test. This is compatible with the cut-off value of ≥21.5 in the original paper by Eloseily et al. [17]. Comparing the 2016 MAS classification criteria with the MS score, we showed consensus in about six patients who met both sets of criteria. There was disagreement about one patient who had a MS score above the cut-off level of -2.1 but did not fulfil the 2016 MAS classification criteria and another patient who fulfilled these criteria but had a MS score below the threshold. Recently, Sag et al. compared the MS-score and the HScore in seventy-one sJIA patients and found that the optimal cut-off for the MS-score in their cohort was ≥ -1.64 with a sensitivity of 91.3% and a specificity of 83.8% [26], being close to the optimal cut-off value at ≥ -1.5 (sensitivity = 71.4%, specificity = 91.7%) that was found in our cohort. The difference between the MS-score ≥ −2.1 presented by Minoia et al. and the cut-off value in our study [16] may depend on selection bias and the small number of patients in our study. In the study by Minoia et al., the median of ferritin in the MAS group was higher (5253 *μ*g/L vs. 3786 *μ*g/L), and the median of platelets was lower (144 × 10^9^/L vs. 190 × 10^9^/L). Among the clinical characteristics, the frequencies of hemorrhage and CNS involvement were higher in the study by Minoia, indicating a more severely affected cohort.

It is an advantage to both the 2016 MAS classification criteria, MS score and the ferritin/ESR ratio that they do not include bone marrow aspiration. This contrasts with the HLH-2004 diagnostic guidelines and the HScore which are more invasive and time-consuming procedures when early diagnosis and treatment of MAS are critical for survival [17]. As the ferritin/ESR ratio only involves two tests, it is the easiest measure to identify MAS compared to the 2016 MAS classification criteria and MS score. However, a common disadvantage with all three sets of criteria is that they relate to a single time point and not changes in laboratory and clinical parameters over time [26]. Many have argued that relative changes in laboratory parameters are more useful for establishing an early MAS diagnosis than a certain threshold [5, 8, 13, 27]. In our study, we found significant changes in ferritin, platelets, WBC, and neutrophils. These results are compatible with findings by Minoia et al. showing an increase in ferritin (556%) and ALT (325%) based on 362 MAS patients from the last visit before MAS until the onset of MAS [8]. Platelet count decreased 55%, which was compatible with our results. Ravelli et al. identified laboratory changes over time based on 115 JIA patients with MAS with an increase in ferritin (819%), AST (379%), LDH (216%), and triglycerides (111%) and a decrease in platelet count (-63%) [5]. Shimizu et al. validated the MAS 2016 criteria in sixty-five Japanese patients with sJIA, showing that ferritin demonstrated the largest change over time indicating its important role in the diagnostics of MAS [28].

Notably, the patients fulfilling all four sets of criteria in the present study all developed recurrent MAS. Genetic evaluations were performed in two of these patients. One patient (ID-18, Table 6) had a LYST-variant and a variant in the NLPR-12 gene. The other patient (ID-24, Table 6), who fulfilled all MAS 2016 criteria, had a rare heterozygous missense variant (R161H) in the *CASP1* gene and in addition a variant in *UNC13D*, as previously reported [10]. We found that the *CASP1* variant is a gain-of-function for both inflammasome and NF-*κ*B activation, leading to increased production of IL-6, IL-1*β*, and IL-18 which may have contributed to the development of MAS [10].

This study has several limitations that need to be addressed. First, the study has a small sample size which limits the statistical power. Second, due to the retrospective study design, some laboratory and clinical data are missing. Some patients were transferred from other hospitals without available laboratory measurements at the initial stages of sJIA. This may have underestimated the subclinical forms of MAS. Third, data on ALT were used instead of AST. This opens the possibility that three of the MAS patients with ALT > 48 U/L could have been misdiagnosed. However, ALT was significantly above its threshold, which may indicate that AST conceivably had also been higher than 48 U/L since both liver parameters have quite similar reference intervals.

## 5. Conclusion

The 2016 MAS classification criteria were a valuable measure discriminating between sJIA with and without MAS in this small cohort, finding a prevalence of MAS in 25% compared to only 9.4% fulfilling the HLH-2004 diagnostic guidelines. All patients identified by the 2016 MAS classification criteria were also captured by the ferritin/ESR ratio exceeding the cut-off point ≥ 21.5, but the specificity was relatively low. In comparison, the MS score had a higher specificity for the identification of MAS. Detection of laboratory alterations may be essential in the early stages of MAS, and even in this small cohort, significant changes in ferritin, platelet, WBC, and neutrophil counts were demonstrable.

## Figures and Tables

**Table 1:** Comparison of HLH-2004 diagnostic guidelines, MAS 2016 classification criteria, MS score, and ferritin to erythrocyte sedimentation rate ratio for the diagnosis of MAS.

	HLH-2004^a^	MAS 2016^b^	MS score^c^	Ferritin:ESR ratio^d^
Laboratory parameters				
Ferritin, *μ*g/L	≥500	>684	Included	Included
Platelets, ×10^9^/L	<100	≤181	Included	—
Aspartate aminotransferase, U/L	—	>48	—	—
Lactate dehydrogenase	—	—	Included	—
Triglycerides, mmol/L	≥3.00	>1.76	—	—
Fibrinogen, *μ*mol/L	≤4.41	≤10.6	Included (mg/dL)	—
Hemoglobin, mmol/L	<5.59	—	—	—
Neutrophils, ×10^9^/L	<1.0	—	—	—
Erythrocyte sedimentation rate, mm	—	—	—	Included
Clinical signs				
Fever	Included	Included	Included	Included
CNS involvement	—	—	Included	—
Splenomegaly	Included	—	—	—
Haemorrhagic manifestations	—	—	Included	—
Arthritis	—	—	Included	—
Others				
Hemophagocytosis in bone marrow or spleen or lymph nodes	Included	—	—	—
Low or absent NK-cell activity	Included	—	—	—
Soluble CD25 (i.e., soluble IL-2 receptor), U/mL	≥2,400	—	—	—
A molecular diagnosis consistent with HLH	Included	—	—	—

^a^HLH-2004 criteria includes molecular diagnosis consistent with HLH or 5 of 8 criteria: fever, splenomegaly, cytopenia affecting ≥ 2 of 3 lineages (Hgb < 5.59 mmol/L, platelets < 100 × 10^9^/L, neutrophils < 1.0 × 10^9^/L), triglycerides ≥ 3.0 mmol/L, fibrinogen ≤ 4.41 *μ*mol/L, hemophagocytosis, low NK-cell activity, ferritin ≥ 500 *μ*g/L, and soluble CD25 ≥ 2,400 U/mL [15]. ^b^MAS 2016 criteria include fever and ferritin > 684 *μ*g/L plus 2 of 4 criteria: platelets ≤ 181 × 10^9^/L, AST > 48 U/L, triglycerides > 1.76 mmol/L, and fibrinogen ≤ 10.6 *μ*mol/L [7]. ^c^Calculation of the MS score: CNS involvement × 2.44 + haemorrhagic manifestations × 1.54 + arthritis × (−1.30) + platelets × (−0.003) + LDH × 0.001 + fibrinogen × (−0.004) + ferritin × 0.0001 [16]. ^d^Ferritin/ESR ratio ≥ 21.5 considered as a screening tool for the diagnosis of MAS [17].

**Table 2:** SJIA patients classified with MAS according to the HLH-2004 diagnostic guidelines, MAS 2016 classification criteria, MS score, and/or ferritin/ESR ratio.

	Recurrent MAS	HLH-2004	MAS 2016^a^	MS score^b^	Ferritin:ESR ratio cut-off point ≥21.5^c^
Patient 1		Not fulfilled	*Fulfilled (3)*	*-1.1*	*53.7*
Patient 10		Not fulfilled	*Fulfilled (2)*	*0.3*	*164.2*
Patient 15^d^		Not fulfilled	*Fulfilled (2)*	*-1.9*	*304.8*
Patient 18	*x*	*Fulfilled*	*Fulfilled (2)*	*-0.8*	*45.6*
Patient 20		Not fulfilled	Not fulfilled	*-0.7*	13.8
Patient 22		Not fulfilled	*Fulfilled (2)*	-2.3	*90.0*
Patient 24	*x*	*Fulfilled*	*Fulfilled (4)*	*3.3*	*481.1*
Patient 30	*x*	*Fulfilled*	*Fulfilled (3)*	*-0.19*	*164.3*
Patient 32		Not fulfilled	*Fulfilled (2)*	-4.4	*25.0*
Fulfilled criteria		*3*/32	*8*/32	**7**/19	*13*/26
Sensitivity (%)^e^		37.5 (8.5-75.5)		75 (34.9-96.8)	100 (63.1-100)
Specificity (%)^e^		100 (85.8-100)		90.9 (58.7-99.7)	72.2 (46.5-90.3)
PPV (%)^e^		100		85.7 (47-97.6)	61.5 (43.2-77.1)
NPV (%)^e^		82.8 (73.7-89.1)		83.3 (59.7-94.4)	100

^a^The number in the parenthesis shows the number of fulfilled MAS criteria other than ferritin > 684 *μ*g/L. It was not possible to evaluate the 2016 MAS criteria in six patients due to missing data on ferritin. ^b^Calculation of the MS score: CNS involvement × 2.44 + haemorrhagic manifestations × 1.54 + arthritis × (−1.30) + platelets × (−0.003) + LDH × 0.001 + fibrinogen × (−0.004) + ferritin × 0.0001. Patients with a MS score ≥ −2.1 are typed in italics. MS score could not be calculated for thirteen SJIA patients due to missing data on ferritin and/or fibrinogen. ^c^Patients with a ferritin: ESR ratio cut point ≥ 21.5 are typed in italics. Ferritin to erytrocyte sedimentation rate ratio could not be calculated for 6 patients due to missing data on both ferritin and ESR. ^d^Patient diagnosed in 2015 developed subclinical MAS 42 days after onset of sJIA. ^e^Sensitivity, specificity, positive predictive value (PPV), and negative predictive value (NPV) calculated against 2016 MAS classification criteria and expressed as percentage with 95% confidence interval in parenthesis.

**Table 3:** Demographic characteristics and laboratory data in children with active SJIA with and without MAS according to the 2016 MAS classification criteria.

	sJIA with MAS	sJIA without MAS	*P* value
Patients	8	24	
Age at onset (years)	11.5 (3.1-15.3)	10.8 (6.4-12.8)	0.761
Gender (f/m)	5/3	9/15	0.217
Ferritin (*μ*g/L)	3786 (2246-8577)	695 (243-2551)	0.006
Platelets (×10^9^/L)	190 (137-272)	425 (317-498)	0.001
Fibrinogen (*μ*mol/L)	10.3 (4.9-10.9)	18.1 (11.4-21.5)	0.008
Triglycerides (mmol/L)	3.0 (1.8-5.8)	1.3 (1.1-2.1)	0.003
ALT (U/L)	128 (20-167)	13 (9-21)	0.006
LDH (U/L)	697 (454-1153)	260 (198-321)	0.003
WBC (×10^9^/L)	7.1 (5.5-11.4)	16.0 (9.2-23.4)	0.007
Neutrophils (×10^9^/L)	4.0 (3.7-4.4)	12.6 (6.1-20.8)	0.002
ESR (mm/hr)	45 (34.8-81.8)	74 (50.8-96)	0.030
CRP (mg/L)	41.3 (10.8-78.9)	104.6 (38.6-134.3)	0.058
Hb (mmol/L)	7.0 (6.0-7.3)	7.0 (5.9-7.3)	0.794
Ferritin/ESR ratio	127.1 (47.6-269.7)	10.5 (4.3-38.3)	<0.001
MS score	-0.95 (-2.2–(+0.18))	- 4.7 (-5.3–(-2.5))	0.003

ALT: alanine aminotransferase; LDH: lactate dehydrogenase; WBC: white blood cells; ESR: erythrocyte sedimentation rate; CRP: C-reactive protein; Hb: hemoglobin. Values are expressed as median with interquartile range in parenthesis. *P* values are obtained using Mann–Whitney *U* test for comparison.

**Table 4:** Laboratory data of patients with sJIA at time of MAS diagnosis according to 2016 MAS criteria.

	Ferritin, *μ*g/L	PLT, ×10^9^/L	Fibrinogen, *μ*mol/L	Triglycerides, mmol/L	ALT, U/L	Number of fulfilled MAS criteria other than high ferritin	Time from SJIA onset to MAS onset (days)
MAS 2016 criteria	Ferritin > 684 *μ*g/L	PLT ≤181 × 10^9^/L	Fibrinogen ≤ 10.6 *μ*mol/L	Triglycerides > 1.76 mmol/L	ALT > 48 U/L		
Patient							
ID-01	*5048*	286	*10.3*	*1.80*	*128*	3	28
ID-10	*2245*	210	*4.8*	*5.3*	24	2	830
ID-15	*9753*	*134*	14.3	1.3	*144*	2	42
ID-18	*2052*	226	10.9	*3.0*	*128*	2	52
ID-22	*4049*	*133*	*10.6*	1.6	9	2	6
ID-24	*20688*	*169*	*4.9*	*7.2*	*380*	4	103
ID-30	*3523*	*146*	*5.5*	*3.1*	18	3	32
ID-32	*2247*	466	15.9	*2.5*	*174*	2	20

PLT: platelets; ALT: alanine aminotransferase. The fulfilled MAS criteria (other than ferritin > 684 *μ*g/L) are in italics text type.

**Table 5:** Changes in laboratory values from last visit before onset of MAS to development of MAS according to 2016 MAS criteria (*n* = 7).

	Normal range	Last visit before MAS	Onset of MAS	*P* value
Ferritin, *μ*g/L	7-152	767 (650-1898)	4049 (2245-9753)	0.018
PLT, ×10^9^/L	135-435	381 (233-578)	210 (134-286)	0.018
ALT, U/L	5-45	12 (10-33)	128 (21-144)	0.128
WBC, ×10^9^/L	4.4-16.2	17.4 (6-23.7)	7.1 (5.4-8.4)	0.043
Neutrophil count, ×10^9^/L	1.20-9.60	13 (4-16.6)	4.1 (3.7-4.5)	0.028
ESR, mm	0-20	60 (28-79)	45 (32-90)	0.866
CRP, mg/L	0-8.0	51.7 (30.1-150.8)	34 (8.2-56.1)	0.128
Hb, mmol/L	6.0-9.9	7.2 (7.0-7.5)	7.0 (6.4-7.4)	0.496

PLT: platelets; ALT: alanine aminotransferase; WBC: white blood cells; ESR: erythrocyte sedimentation rate; CRP: C-reactive protein; Hb: hemoglobin. Values are given as median with interquartile range in parenthesis. *P* values are obtained using Wilcoxon signed rank test for paired comparison.

**Table 6:** Sensitivity and specificity of components of 2016 MAS classification criteria.

	SJIA with MAS	SJIA without MAS	*P* value	Sensitivity (%)	Specificity (%)	Positive predictive value (%)	Negative predictive value (%)
Ferritin > 684 *μ*g/L	8/8	10/18	0.023	100 (63.1-100)	44.4 (21.5-69.2)	44.4 (34.6-54.7)	100
PLT ≤ 181 × 10^9^/L	4/8	1/24	0.002	50 (15.7-84.3)	95.8 (78.9-99.9)	80 (34.2-96.9)	85.2 (74.1-92.0)
Fibrinogen ≤ 10, 6 *μ*mol/L	5/7	2/11	0.024	71.4 (29-96.3)	81.8 (48.2-97.7)	71.4 (39.6-90.5)	81.8 (57.5-93.8)
Triglycerides > 1.76 mmol/L	6/7	3/12	0.011	85.7 (42.1-99.6)	75 (42.8-94.5)	66.7 (41.8-84.8)	90 (58.8-98.3)
ALT > 48 U/L	5/8	3/24	0.005	62.5 (24.5-91.5)	87.5 (67.6-97.3)	62.5 (33.7-84.5)	87.5 (73.9-94.6)

PLT: platelets; ALT: alanine aminotransferase. Sensitivity, specificity, positive predictive values, and negative predictive values are expressed as percentages with 95% confidence interval in parenthesis. *P* values are obtained using Pearson chi-square test.

## Data Availability

Data used to support the findings of this study are included within the article.
